# Research on the Intervention of Bile Stasis-Induced Pruritus Using Small Molecules Targeting MRGPRX4 Through High-Throughput Screening

**DOI:** 10.3390/molecules31203635

**Published:** 2026-10-12

**Authors:** Qi Liang, Jiahao Kuang, Liangchen Wang, Hanghang Gou, Dandan Tang, Wei Peng, Chunjie Wu

**Affiliations:** 1 Chinese Medicine Germplasm Resources Innovation and Effective Uses Key Laboratory of Sichuan Province, School of Pharmacy, School of Modern Chinese Medicine Industry, Institute of Innovation, Chengdu University of Traditional Chinese Medicine, Chengdu 611137, China; liangqi@stu.cdutcm.edu.cn (Q.L.); 2026zs883@stu.cdutcm.edu.cn (J.K.); wangliangchen@stu.cdutcm.edu.cn (L.W.); ghh@stu.cdutcm.edu.cn (H.G.); 2 School of Pharmacy, Sichuan College of Traditional Chinese Medicine, Mianyang 621000, China

**Keywords:** MRGPRX4, virtual screening, cholestasis-induced itching, small-molecule inhibitor

## Abstract

MRGPRX4, which is mainly expressed in human sensory neurons, is a key mediator of cholestatic pruritus, but clinically available small-molecule inhibitors targeting it remain very scarce; thus, this study aims to screen out novel active inhibitors targeting MRGPRX4. In this work, we performed high-throughput virtual screening of the small-molecule compound library with Schrodinger software targeting MRGPRX4, and selected the top five commercially available compounds with the highest scores from 13,144 candidate compounds. Molecular docking and molecular dynamics simulation were used for virtual validation, and the results confirmed that all five compounds could bind to MRGPRX4, among which 6′-O-malonyldaidzin showed the most favorable binding free energy and a compact binding pocket with the lowest SASA value. We further verified the effect of these compounds on Ca^2+^ influx in 50 μM concentration in HEK293 cells overexpressing MRGPRX4, and confirmed the inhibitory activity of 6′-O-malonyldaidzin, whose binding interaction with MRGPRX4 was additionally verified by CETSA. In conclusion, this study identified 6′-O-malonyldaidzin as a promising lead compound for further pharmacological characterization and lead optimization, with potential for development as a targeted intervention for cholestatic pruritus.

## 1. Introduction

Chronic itching is a widespread and debilitating condition that significantly impairs the quality of life of patients and constitutes a significant unmet medical need. Unlike acute itching, which is a protective response, chronic itching can persist for weeks or months without external stimuli and often resists traditional antihistamine drugs or corticosteroid treatments [1,2]. It typically occurs in the context of systemic diseases, including cholestatic liver diseases, renal dysfunction, and neurological disorders, but the molecular mechanism remains unclear [3,4]. Particularly for cholestatic itching, which is common in patients with primary biliary cholangitis and pregnancy-induced intrahepatic cholestasis [5], the existing treatment drugs such as ursodeoxycholic acid, masoproterone, and rifampicin have limited efficacy, and new targeted intervention methods are urgently needed [6,7,8]. Recent research findings have shown that MRGPRX4 is mainly expressed in human sensory neurons and is a key mediator of cholestasis-induced itching [9,10,11]. Due to the lack of effective selective antagonists or inverse agonists, the therapeutic targeting of MRGPRX4 has always been challenging.

Virtual screening, due to its advantages such as high efficiency, low cost, and the ability to quickly focus on the chemical framework, has become an important technical means in early drug discovery [12,13]. Combined with the CETSA (Cell-based Thermal Shift Assay) technology, it can further verify the direct interaction between the candidate compounds and the target protein at the biophysical level, and combine molecular docking, cell experiments, and drugability prediction to achieve multi-dimensional evaluation of the candidate molecules [14,15,16,17,18]. Based on this, in this study, targeting MRGPRX4, virtual screening was used to screen potential binding molecules from the compound library, and through molecular docking, molecular dynamics simulation, Ca^2+^ flow detection, CETSA, and absorption, distribution, metabolism, excretion and toxicity (ADMET) prediction, a systematic analysis of the candidate compounds was conducted, aiming to discover potential inhibitors and provide experimental basis for the further optimization and development of targeted drugs for cholestasis-induced pruritus.

## 2. Results

### 2.1. Top 5 Compounds Discovered Based on Virtual Screening

**Figure 1:**
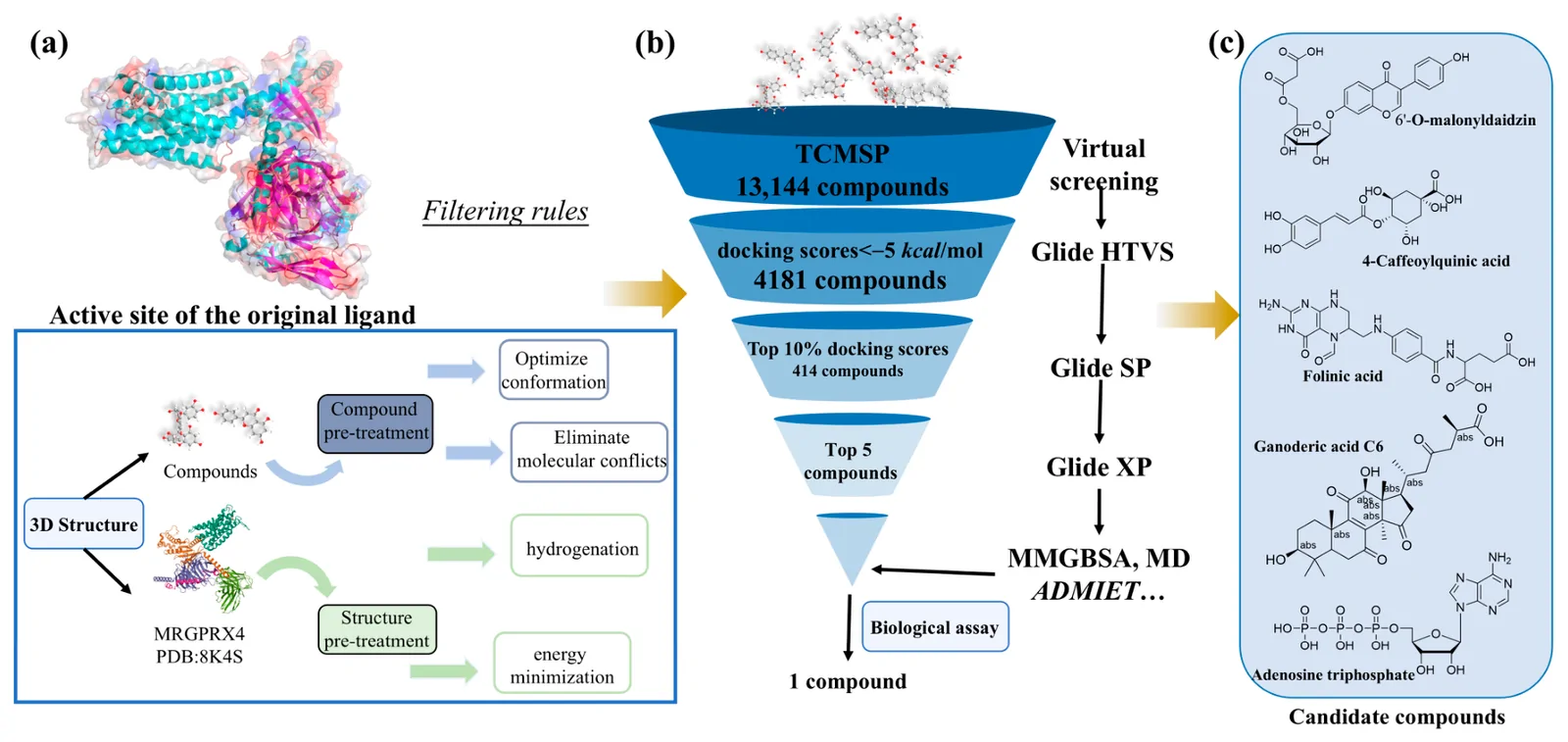
Workflow of hierarchical virtual screening for MRGPRX4 inhibitors. (**a**) Schematic overview of 3D structure preprocessing. (**b**) The sequential virtual screening funnel. (**c**) Chemical structures of the top five candidate compounds obtained through virtual screening.

To identify MRGPRX4 inhibitors, 13,144 compounds from the Traditional Chinese Medicine Systems Pharmacology (TCMSP) database were virtually screened using the Glide module in Schrödinger Maestro 11.5. First, all 13,144 compounds were subjected to molecular docking using the Glide High-Throughput Virtual Screening (HTVS) protocol, and compounds with a docking score of <−5 kcal/mol (4181 compounds) were subsequently subjected to Standard Precision (SP) docking. The top 10% of compounds (414 compounds) from the SP stage were then further evaluated using Extra Precision (XP) docking. From the XP output, the top 10% of compounds (based on Glide XP G-score) were retained, and these were rescored using the MM/GBSA method to estimate binding free energy (ΔGbind). The final five compounds were then selected according to the following combined criteria: ① favorable Glide XP docking score, ② favorable MM/GBSA binding free energy, ③ commercial availability, and ④ structural diversity. Accordingly, 6′-O-malonyldaidzin, 4-caffeoylquinic acid, folinic acid, ganoderic acid C6, and adenosine triphosphate (ATP) were selected for subsequent molecular dynamics simulations and binding free energy calculations. These compounds were 6′-O-malonyldaidzin, 4-caffeoylquinic acid, folinic acid, ganoderic acid C6, and adenosine triphosphate (ATP) (Figure 1a–c).

**Figure 2:**
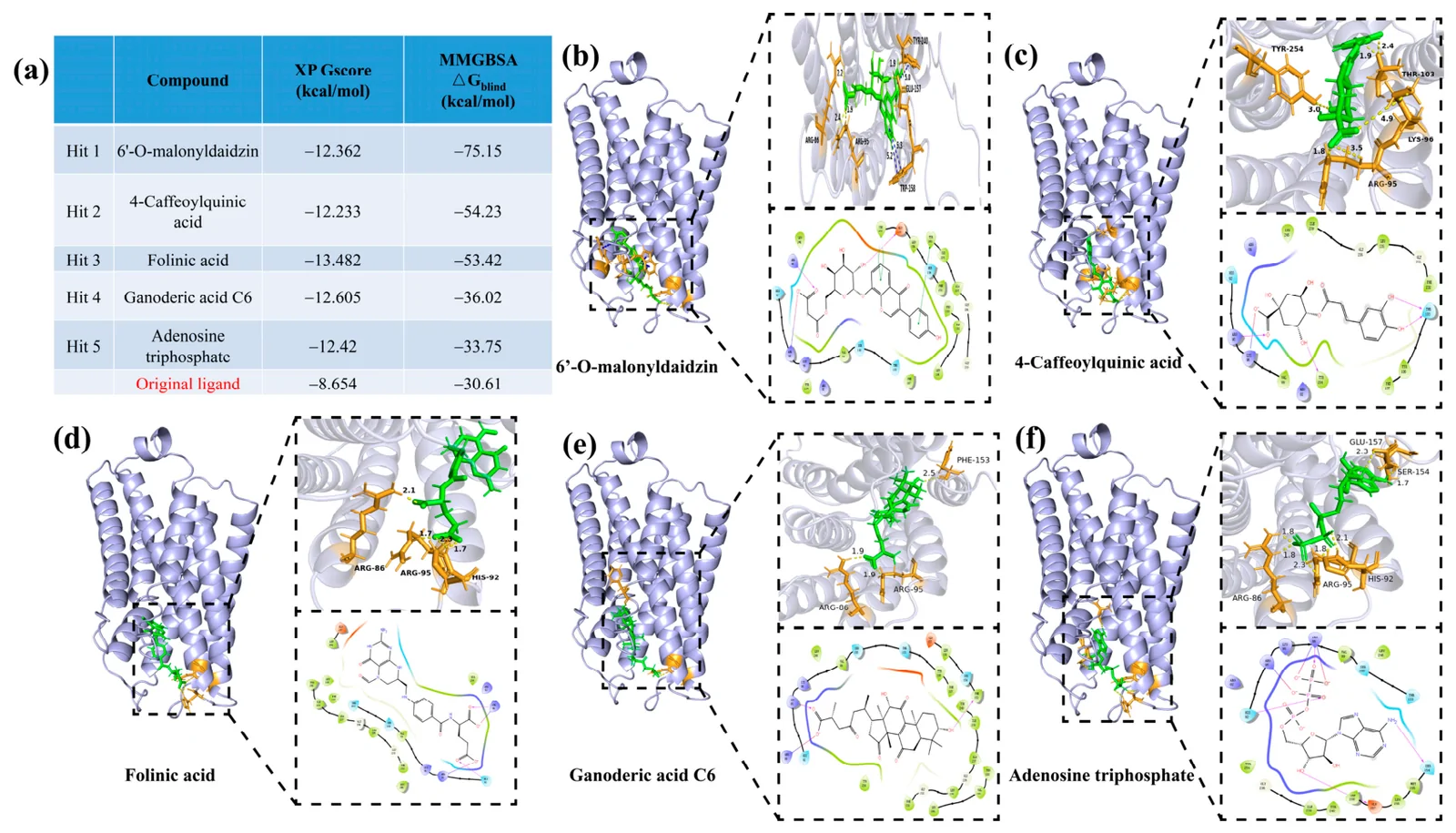
Docking scores and binding modes of the top five candidates against MRGPRX4. (**a**) Glide XP G-score and MMGBSA-calculated binding free energy (ΔGbind) of five candidates and the original co-crystallized ligand of MRGPRX4. (**b**–**f**) 3D binding mode (upper right panel) and 2D ligand–residue interaction diagram (lower right panel) of 6′-O-malonyldaidzin (**b**), 4-caffeoylquinic acid (**c**), folinic acid (**d**), ganoderic acid C6 (**e**), and adenosine triphosphate (**f**) in the MRGPRX4-binding pocket, respectively. (Purple Arrows: Hydrogen Bond; Green Lines: π-π Stacking; Red Lines: π-Cation Interaction; Red-Blue Lines: Salt Bridge) Key hydrogen bonding interactions are marked with their corresponding bond lengths.

The calculated binding free energies for 6′-O-malonyldaidzin, 4-caffeoylquinic acid, folinic acid, ganoderic acid C6, and ATP were −75.15, −54.23, −53.42, −36.02, and −33.75 kcal/mol, respectively (Figure 2a). The molecular docking poses of these five compounds were subsequently visualized using PyMOL 3.0.3 (Figure 2b–f). 6′-O-Malonyldaidzin formed two direct hydrogen bonding interactions: one between its succinate carboxylate group and ARG 95, and another between the carboxylate and ARG 86. In addition, two π–π stacking interactions were observed between the two aromatic rings of the ligand and active site residues TRP 158 and TYR 240, respectively. 4-Caffeoylquinic acid displayed hydrogen bonds where the carboxylate group interacts with both ARG 95 and LEU 96, the 1-hydroxyl group forms a bond with THR 254, and the two adjacent phenolic hydroxyl groups on the caffeoyl moiety both form hydrogen bonds with THR 103. Folinic acid formed two distinct hydrogen bonds via its two terminal carboxylate groups with ARG 96 and ILE 92, anchoring the ligand in the binding pocket. Ganoderic acid C6 showed a carboxylate group on the side chain forming two hydrogen bonds with ARG 86 and ARG 95, while the terminal hydroxyl group on the steroidal skeleton formed an additional hydrogen bond with TYR 240 [19,20]. ATP formed an extensive hydrogen-bonding network with multiple positively charged active site residues, including interactions with ARG 86, LYS 96, ARG 95, and LYS 92. The 2′-hydroxyl group on the ribose formed an additional hydrogen bond with TYR 158, and the 6-amino group on the adenine aromatic ring formed a hydrogen bond with SER 154.

**Figure 3:**
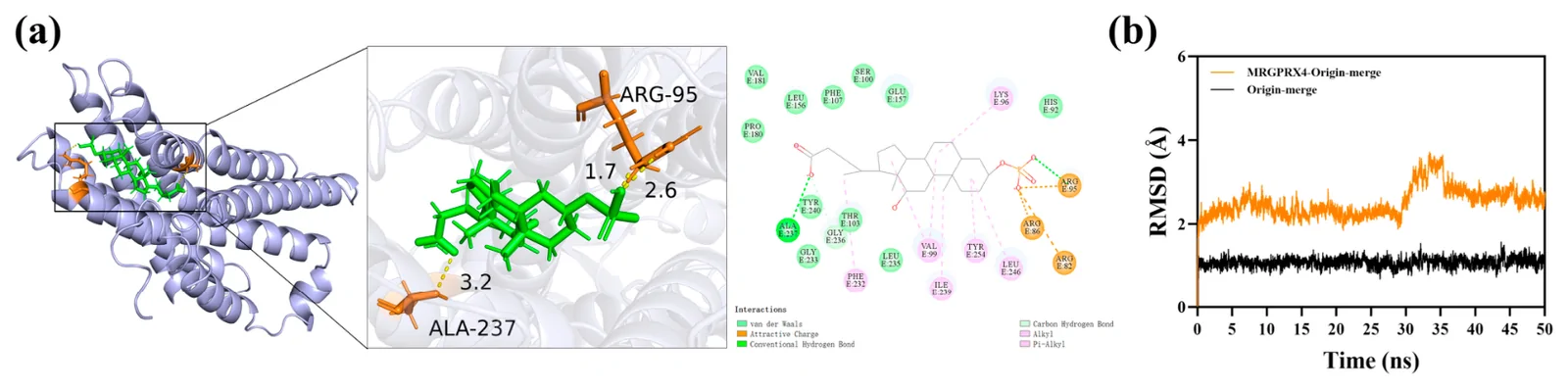
(**a**,**b**) Docking validation by redocking the co-crystallized ligand (PDB 8K4S) into the MRGPRX4-binding site. The 2D interaction map shows key intermolecular contacts; heavy-atom RMSD = 2.7 Å between redocked and experimental poses.

The ARG95 and ALA237 residues on the MRGPRX4 receptor form hydrogen bond interactions with origin-merge. The LYS96, LEU246, TYR254, ILE239, VAL99 and PHE232 residues on the MRGPRX4 receptor form hydrophobic interactions with origin-merge. The HIS92, LEU157, SER100, PHE107, LEU156, VAL181, PRO180, TYR240, THR103, GLY233 and LEU235 residues on the MRGPRX4 receptor form van der Waals interactions with origin-merge. The GLY236 residue on the MRGPRX4 receptor forms carbon–hydrogen bond interactions with origin-merge. The ARG95, ARG86 and ARG82 residues on the MRGPRX4 receptor form attractive charge interactions with origin-merge. To validate the reliability of the docking protocol, the co-crystallized ligand from PDB 8K4S was redocked into the MRGPRX4-binding site using the same Glide XP parameters. The predicted pose was compared with the original co-crystal pose, yielding a heavy-atom RMSD of 2.7 Å (Figure 3a,b), which indicates acceptable reproduction of the experimental binding mode and supports the credibility of the structure-based virtual screening workflow.

### 2.2. Molecular Dynamics Simulation

Molecular dynamics (MD) simulations were performed to evaluate the interaction stability of the five candidate compounds with MRGPRX4 [21,22,23,24]. The results are organized according to the molecular property assessed.

**Figure 4:**
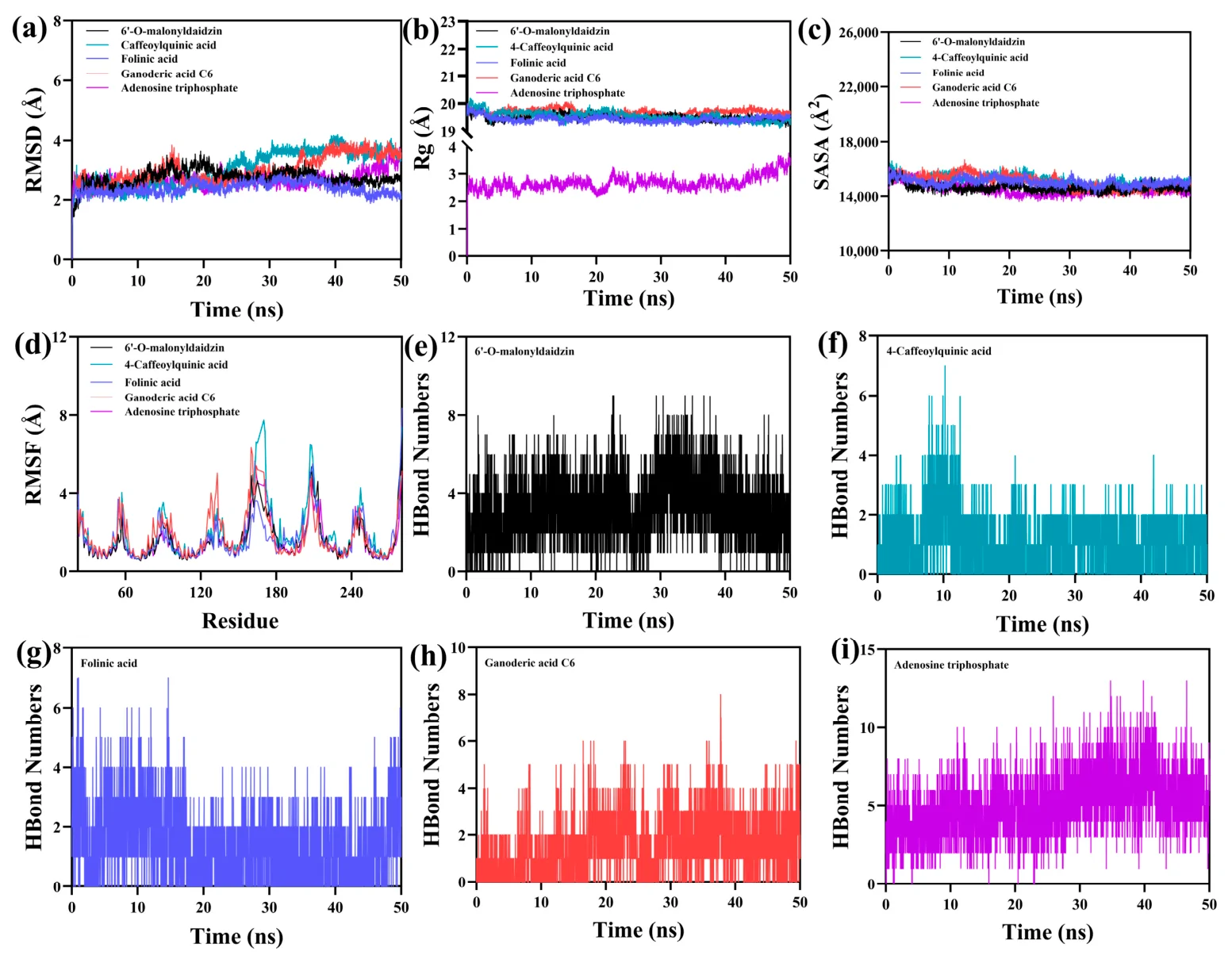
Conventional analysis of 50 ns molecular dynamics simulations for MRGPRX4–candidate complexes. (**a**) Root mean square deviation (RMSD) of Cα atoms of MRGPRX4 in five independent complex systems. (**b**) Radius of gyration (Rg) of MRGPRX4 to reflect overall structural compactness. (**c**) Solvent accessible surface area (SASA) of the MRGPRX4-binding pocket. (**d**) Root mean square fluctuation (RMSF) of individual residues of MRGPRX4 to assess local flexibility. (**e**–**i**) The number of stable hydrogen bonds between MRGPRX4 and 6′-O-malonyldaidzin (**e**), 4-caffeoylquinic acid (**f**), folinic acid (**g**), ganoderic acid C6 (**h**), and adenosine triphosphate (**i**) during simulation, respectively.

(1) Receptor conformational stability: Protein Cα RMSD analysis (Figure 4a) indicated that all five MRGPRX4–ligand complexes reached equilibrium after 25–45 ns and maintained stable conformations throughout the 50 ns simulation. The equilibrium fluctuations were as follows: MRGPRX4–folinic acid (2.39 Å), MRGPRX4–ganoderic acid C6 (3.00 Å), MRGPRX4–4-caffeoylquinic acid (3.03 Å), MRGPRX4–6′-O-malonyldaidzin (2.73 Å), and MRGPRX4–adenosine triphosphate (2.67 Å). Among the five, the MRGPRX4–folinic acid complex exhibited the lowest protein Cα RMSD, whereas 6′-O-malonyldaidzin showed the second lowest. These values reflect the overall stability of the receptor backbone rather than direct ligand binding mode stability. It should be noted that, given the 50 ns simulation timescale, these observations support conformational stability over the simulated interval but do not establish long-term complex stability. Radius of gyration (Rg) analysis (Figure 4b) showed that four complexes exhibited slight fluctuations within the range of 19–20 Å, indicating well-maintained receptor compactness during the simulations.

(2) Local residue fluctuation: RMSF analysis (Figure 4d,e) revealed that the majority of protein residues in all complexes had low flexibility (RMSF < 3 Å), suggesting a high degree of conformational stability in the receptor structure.

(3) Solvent exposure of the binding region: SASA analysis (Figure 4c) focused on the MRGPRX4-binding pocket. All five complexes showed minor fluctuations in solvent-accessible surface area, indicating that binding of the small molecules altered the local microenvironment and led to slight changes in pocket exposure.

(4) Ligand binding mode stability: Ligand RMSD (calculated for heavy atoms after fitting to the protein) showed that 6′-O-malonyldaidzin, 4-caffeoylquinic acid, folinic acid, ganoderic acid C6, and ATP exhibited mean deviations of 2.3 Å, 1.6 Å, 4.3 Å, 2,3 Å, 2.0 Å, respectively, suggesting stable retention of their initial docking poses.

(5) Persistence of hydrogen bonds: Hydrogen bond analysis (Figure 4e–i) showed that the number of hydrogen bonds between each ligand and MRGPRX4 fluctuated between 0 and 7, with most complexes maintaining approximately 3 hydrogen bonds on average. This indicates that this ligand has a strong hydrogen bond interaction with the target protein.

**Figure 5:**
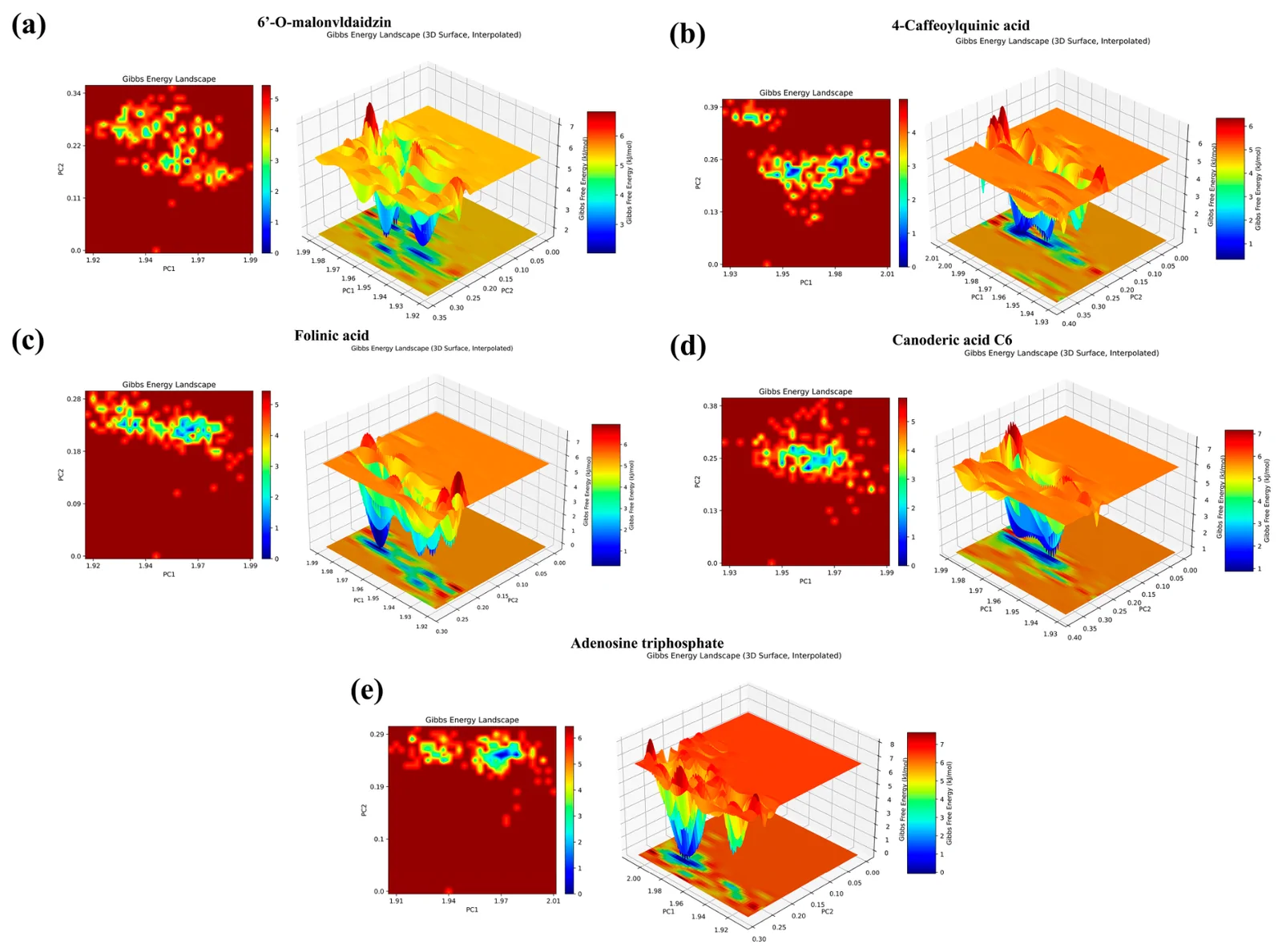
Gibbs free energy landscape (FEL) of MRGPRX4–candidate complexes after principal component analysis. (**a**) 6′-O-malonyldai**d**zin complex, (**b**) 4-caffeoylquinic acid complex, (**c**) folinic acid complex, (**d**) ganoderic acid C6 complex, (**e**) adenosine triphosphate complex. Both 2D contour plots and 3D interpolated surface plots of free energy are presented, and blue regions represent low-energy (conformationally stable) clusters of the complex.

Free energy landscape (FEL) analysis (Figure 5a–e) revealed well-defined low-energy conformational regions in each complex: MRGPRX4–folinic acid (PC2 = 0.20, PC1 = 1.975), MRGPRX4–ganoderic acid C6 (PC2 = 0.20, PC1 = 1.965), MRGPRX4–4-caffeoylquinic acid (PC2 = 0.23, PC1 = 1.990), MRGPRX4–6′-O-malonyldaidzin (PC2 = 0.24, PC1 = 1.950), and MRGPRX4–adenosine triphosphate (PC2 = 0.22, PC1 = 1.995). These low-energy clusters indicate stable conformational states.

Overall, the MD results demonstrate that folinic acid, ganoderic acid C6, 4-caffeoylquinic acid, 6′-O-malonyldaidzin, and adenosine triphosphate all form stable complexes with MRGPRX4, characterized by stable receptor conformations, compact binding pockets, and persistent hydrogen bond interactions. Among them, folinic acid exhibited the lowest protein Cα RMSD, whereas 6′-O-malonyldaidzin showed the lowest SASA value and favorable ligand binding mode stability and was identified as the most promising bioactive candidate.

### 2.3. In Silico Physicochemical Property and ADMET Prediction of the Five Compounds

In order to preliminarily evaluate the drug potency of the five compounds, in silico ADMET prediction analysis was performed on the five candidate compounds using ADMETlab 3.0 [25]. The computational predictions showed that all five compounds predicted human intestinal absorption (HIA) levels ≥ 30%, suggesting that they may have certain absorption potential. However, the predicted plasma protein binding of the candidate compounds was 28.09–76.79%, which was lower than 90%, suggesting that their high proportion of free drug concentration in blood may be conducive to the development of drug effect. Meanwhile, its predicted half-life ranged from 1.12 to 2.58 h, suggesting a potentially long duration of action in vivo. It should be emphasized that these are computational predictions rather than experimentally determined pharmacokinetic parameters. ADMETlab 3.0 prediction can provide a reference for early structural optimization, but it is not yet possible to directly prove that candidate compounds have good in vivo pharmacokinetic properties. It still needs to be further verified by in vitro metabolic stability, plasma protein binding rate and in vivo pharmacokinetics experiments (Table 1).

**Figure 6:**
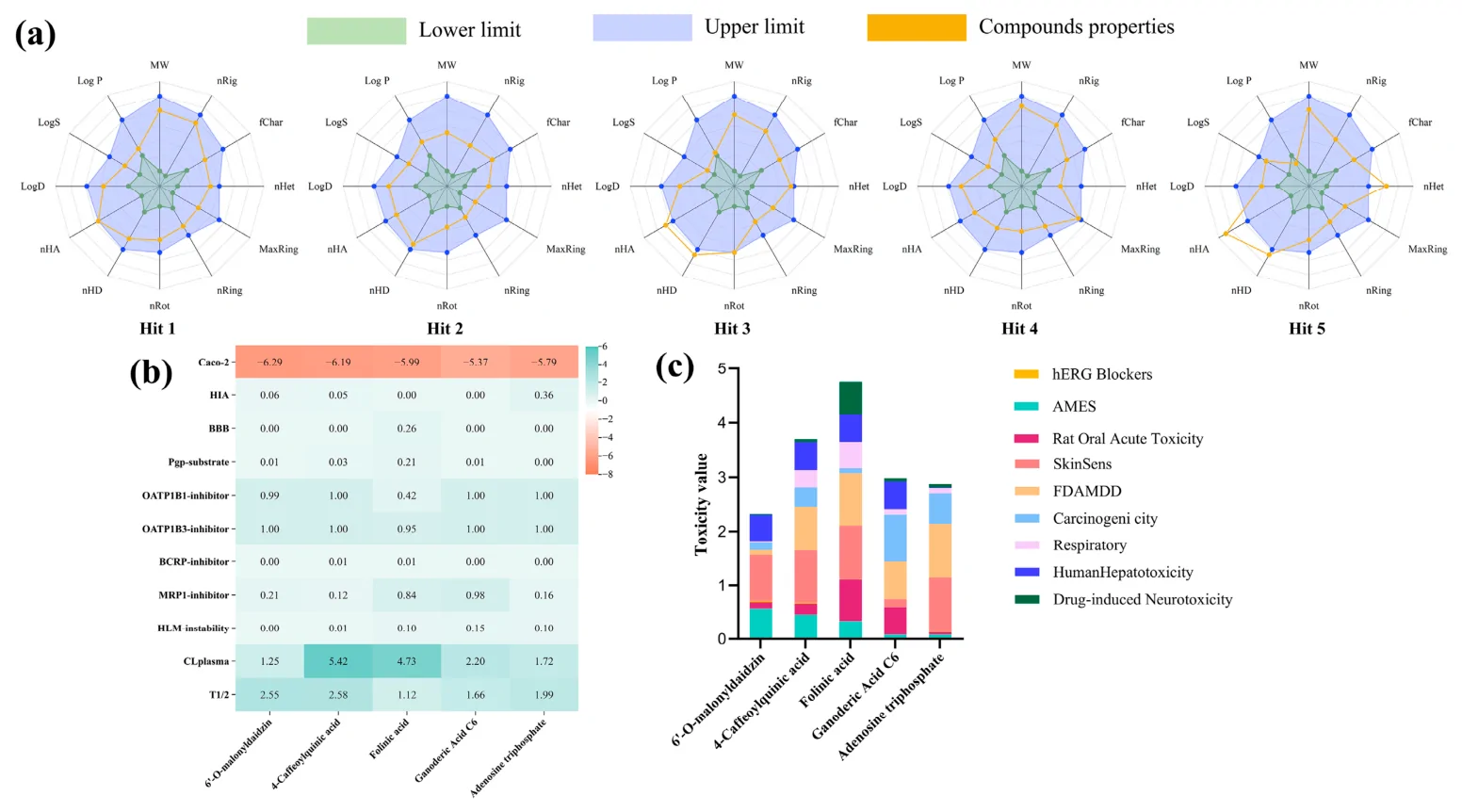
Drug-likeness and ADMET (absorption, distribution, metabolism, excretion, toxicity) prediction of five candidate compounds. (**a**) Radar plots comparing key physicochemical properties of candidates with the acceptable range for oral drug-likeness, including molecular weight (MW), lipophilicity, hydrogen bond donors/acceptors, rotatable bonds, and other topological descriptors. (**b**) Predicted core ADMET properties of the five candidates, covering intestinal absorption, transporter inhibition, metabolic stability, and pharmacokinetic parameters. (**c**) Stacked bar plot summarizing predicted toxicity profiles of five candidates across multiple clinically relevant toxicity endpoints.

To evaluate the developability of the five bioactive candidate hits obtained through virtual screening, we systematically predicted their drug-likeness, ADMET characteristics and toxicological profiles (Figure 6).

As shown in Figure 6a, the drug-likeness radar plot illustrated that all core physicochemical descriptors (including molecular weight, hydrogen bond donors/acceptors, lipophilicity, solubility and structural parameters) of the five candidates fell within the acceptable range of standard drug-likeness guidelines, indicating that all candidates meet the basic requirements for oral lead drug development. Only the number of hydrogen bond acceptors of Hit 5 (adenosine triphosphate) was slightly close to the lower limit of the acceptable range, while all parameters of the other four candidates were located in the ideal interval.

ADMET prediction results (Figure 6b) showed that three candidates (6′-O-malonyldaidzin, 4-caffeoylquinic acid, adenosine triphosphate) had high predicted human intestinal absorption, while folinic acid and ganoderic acid C6 presented low HIA scores. All five candidates had no predicted blood–brain barrier penetration capacity, which is a desirable feature that can reduce the risk of central nervous system side effects for our peripheral target. All candidates showed negative prediction for BCRP inhibition, while all presented positive prediction for OATP1B1 and OATP1B3 inhibition, indicating that transporter-related drug–drug interactions need to be considered in follow-up research. All candidates had low predicted risk of human liver microsome instability, and 4-caffeoylquinic acid and folinic acid showed more appropriate plasma clearance and half-life, indicating more desirable intrinsic pharmacokinetic characteristics.

Toxicity risk assessment (Figure 6c) demonstrated that the total toxicity score (higher value indicates higher toxicity risk) ranked as follows: folinic acid (≈4.8) > 4-caffeoylquinic acid (≈3.7) > ganoderic acid C6 (≈2.9) > adenosine triphosphate (≈2.8) > 6′-O-malonyldaidzin (≈2.4). 6′-O-malonyldaidzin had the lowest overall toxicity, with only low-level risk of AMES mutagenicity and hERG blockade; adenosine triphosphate also presented a low-toxicity profile, with only weak skin sensitization and hERG blockade risk. In general, the main toxicity risks of all five candidates derived from hERG blockade, skin sensitization and hepatotoxicity, and only folinic acid carried an additional drug-induced neurotoxicity risk. The overall toxicity of all five candidates was within the acceptable range for lead compound development.

In conclusion, all five candidate hit compounds identified in this study conform to basic drug-likeness criteria based on in silico predictions, and exhibit acceptable potentially favorable ADMET characteristics and low predicted toxicity risk. They are suitable for further in vitro/vivo activity verification and structural optimization as lead compounds. However, it should be noted that these are preliminary computational predictions that require experimental validation.

### 2.4. MRGPRX4 mRNA Was Markedly Increased in pSE8284-Infected HEK293 Cells

**Figure 7:**
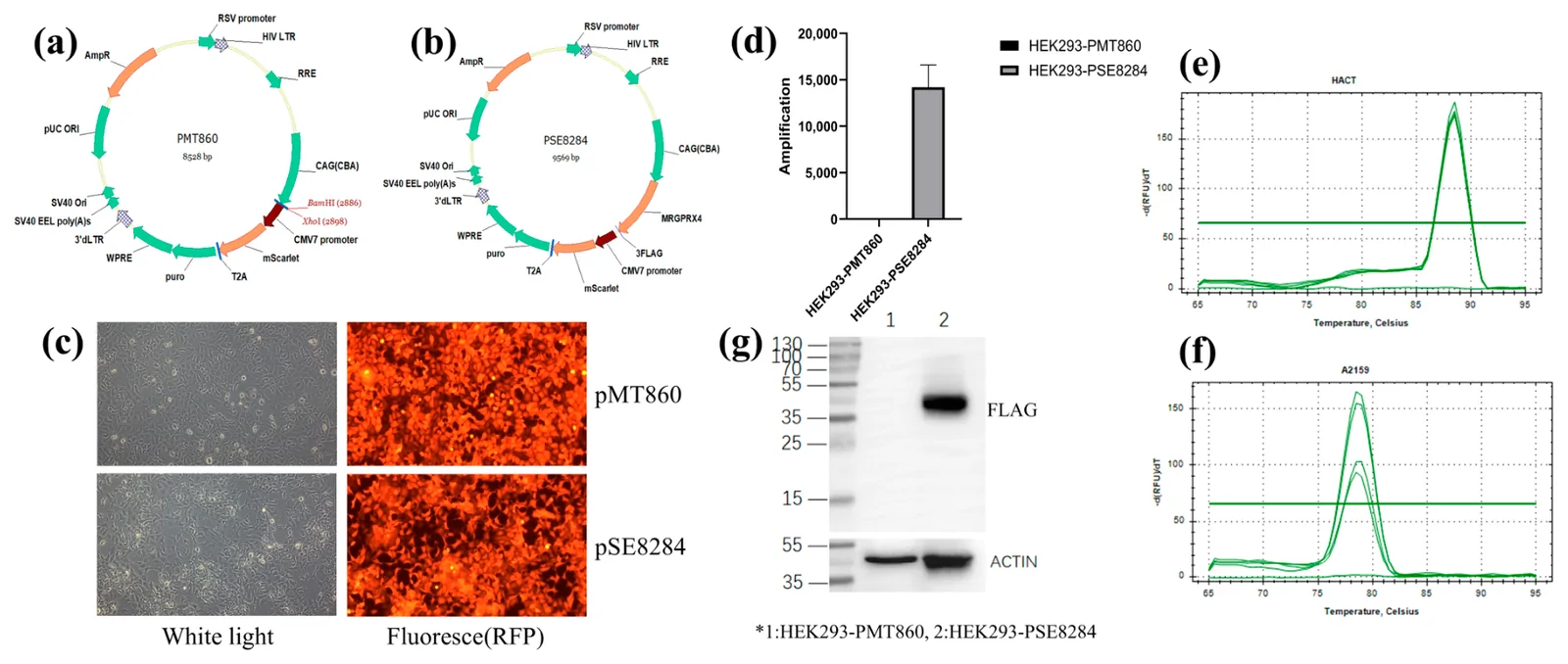
Construction and identification of HEK293 cell line stably overexpressing MRGPRX4. (**a**) Schematic map of the empty control plasmid PMT860. (**b**) Schematic map of the recombinant MRGPRX4 expression plasmid PSE8284. (**c**) Brightfield (white light) and RFP fluorescence imaging of antibiotic-selected stable cell lines. (**d**) Quantitative amplification of MRGPRX4 in the control (HEK293-PMT860) and MRGPRX4 overexpression (HEK293-PSE8284) stable cell lines. (**e**,**f**) qPCR melting curves for amplification of target/reference genes, verifying amplification specificity. (**g**) Western blot validation of FLAG-tagged MRGPRX4 protein expression, with β-actin as the loading control. *1: HEK293-PMT860; 2: HEK293-PSE8284.

The results showed that the MRGPRX4 overexpression vector pSE8284 was successfully constructed. After colony PCR screening and Sanger sequencing verification, the target gene MRGPRX4 and 3FLAG sequences were correctly inserted into the expression vector. Subsequently, HEK293 cells were infected with the pSE8284 lentivirus and screened by 2 μg/mL puromycin for 2 weeks, and HEK293 cell lines with stable expression of MRGPRX4 were obtained, suggesting that the stable MRGPRX4 overexpression strain was successfully constructed (Figure 7a–c).

Quantitative real-time PCR was performed to verify the overexpression efficiency of MRGPRX4 in HEK293 stable cells. Compared with the control lentivirus group HEK293-pMT860, the MRGPRX4-overexpressing group HEK293-pSE8284 showed a marked increase in MRGPRX4 mRNA expression. Based on the 2-ΔΔCt analysis, MRGPRX4 expression was increased by approximately 14,200-fold in HEK293-pSE8284 cells compared with HEK293-pMT860 cells. Melting curve analysis showed no abnormal peaks, indicating specific amplification without obvious primer-dimer formation or nonspecific products (Figure 7d–f). The qPCR results are summarized below (Table 2).

Western blot analysis was further performed using an anti-FLAG antibody to detect FLAG-tagged MRGPRX4 protein. The theoretical molecular weight of MRGPRX4 is approximately 36 kDa. Compared with HEK293-pMT860 control cells, HEK293-pSE8284 cells showed a markedly stronger FLAG-positive band at the expected molecular weight, indicating successful expression of FLAG-tagged MRGPRX4 protein (Figure 7g). Together, the qPCR and Western blot results confirmed that the MRGPRX4-overexpressing HEK293 stable cell line was successfully established.

### 2.5. Evaluation of the Activity of the Five Compounds Obtained by Virtual Screening

**Figure 8:**
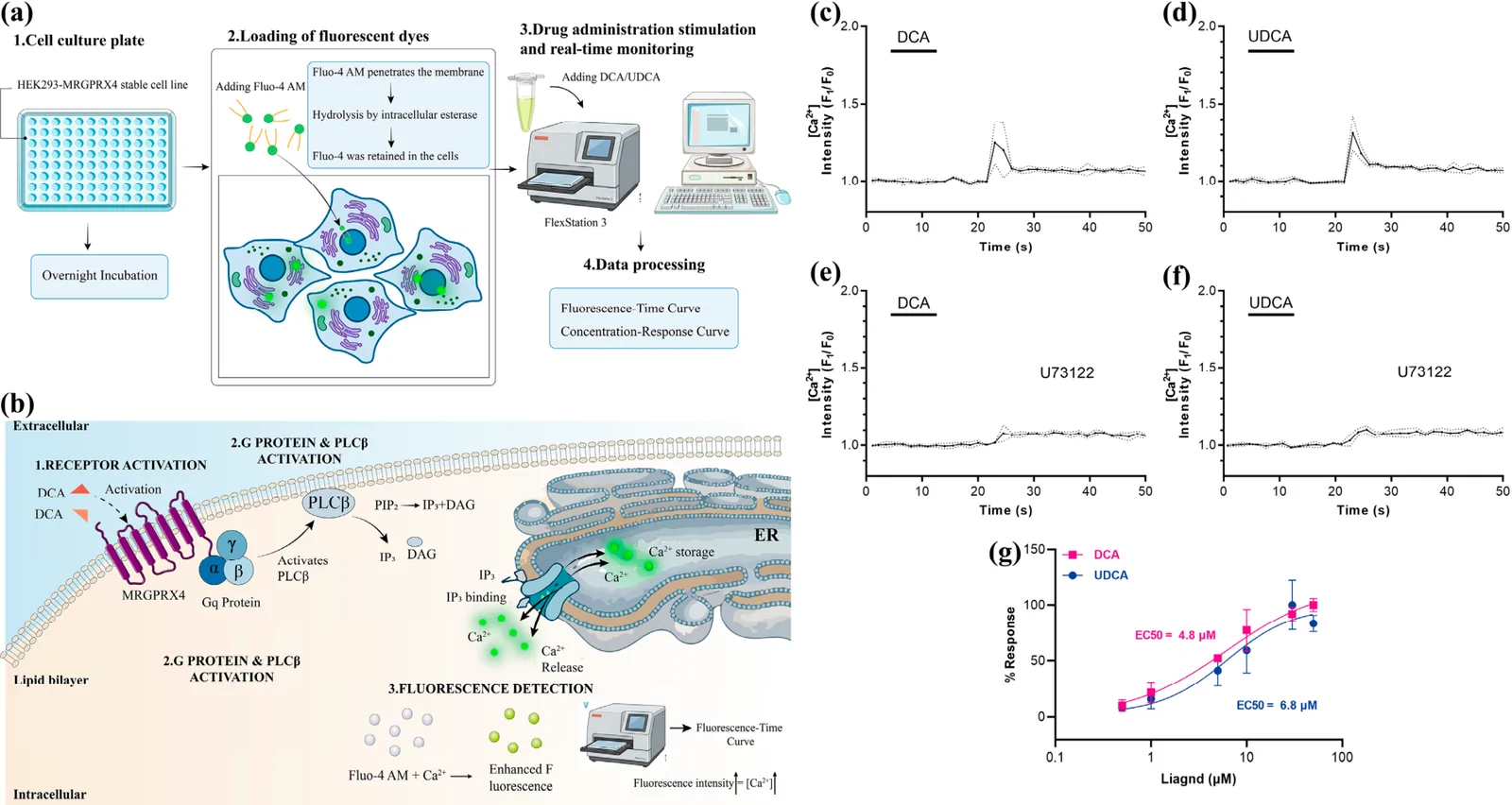
DCA and UDCA trigger MRGPRX4-mediated intracellular calcium signaling via Gq-PLC pathway. (**a**) Workflow of the Fluo-4 AM-based intracellular calcium flux assay. (**b**) Schematic diagram of the MRGPRX4 downstream signaling (**c**,**d**) Representative calcium response traces after stimulation with 10 μM DCA (**c**) or UDCA (**d**) at 20 s. (**e**,**f**) Representative calcium response traces after 30 min pre-incubation with the PLC inhibitor U73122 before DCA (**e**) or UDCA (**f**) stimulation. (**g**) Concentration–response curves of DCA and UDCA for MRGPRX4 activation, with fitted EC_50_ values.

It has been reported that DCA, a potent endogenous agonist of MRGPRX4, induces Ca^2+^ via the Gq pathway [26]. DCA-induced Ca^2+^ influx was attenuated in the presence of inhibitors, as reflected by a decrease in fluorescence intensity in the Flex Station 3 assay (Figure 8a,b). Both DCA and UDCA were tested to stimulate the release of relevant Ca^2+^ signals in HEK293 cells stably expressing MRGPRX4 (Figure 8c,d). In addition, the inhibitor U73122 was shown to inhibit the Ca^2+^ signal (Figure 8e,f). Moreover, the EC50 of DCA and UDCA is about 4.8μM and 6.8μM, respectively (Figure 8g).

**Figure 9:**
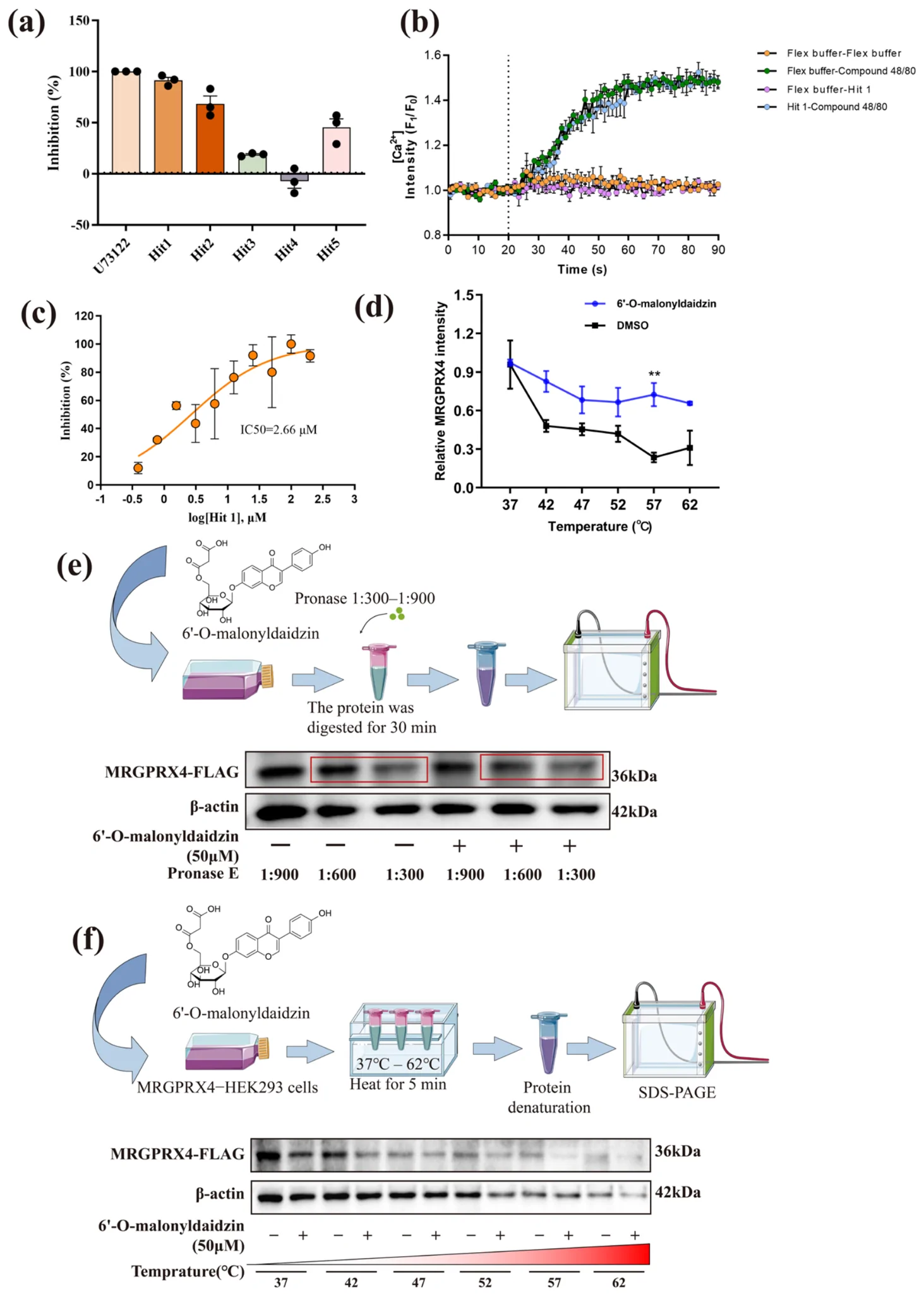
Identification of 6′-O-malonyldaidzin as a direct MRGPRX4 inhibitor. (**a**) Inhibition rates of 50 μM Hit1 to Hit5 candidate compounds against DCA-induced MRGPRX4 activation. (**b**) Selectivity validation: Hit 1 does not trigger MRGPRX2-mediated calcium response, confirming no off-target activation. (**c**) Dose–response curve of Hit 1 (6′-O-malonyldaidzin) for MRGPRX4 inhibition. (**d**) Relative MRGPRX4 activity across gradient temperature in CETSA, with or without 6′-O-malonyldaidzin treatment. (**e**) DARTS. (**f**) CETSA. (Data in panels (**d**,**e**) are presented as mean ± SEM from three independent experiments. ** *p* < 0.01 vs. DMSO).

Then, HEK293 cells stably expressing MRGPRX4 were stimulated with 10 μM DCA, and compounds Hit 1–5 (all 50 μM) were screened to identify MRGPRX4 inhibitors. The results showed that compounds Hit 1, Hit 2, Hit 3, and Hit 5 had inhibitory effects on MRGPRX4. The inhibition rates were 91%, 68%, 19%, and 45%, with compound Hit 4 showing no inhibitory effect on MRGPRX4 and compound Hit 1 showing the best inhibitory effect (Figure 9a).

Importantly, Hit 1 showed minimal cross-reactivity with other itch-related MRGPRX family members. Specifically, Hit 1 neither activates nor inhibits MRGPRX2, highlighting its relatively selective for MRGPRX4 in the itch-related MRGPRX receptor subfamily (Figure 9b). To further investigate the inhibitory effect of Hit 1, the active molecule identified in the initial screening, the final concentration gradients of 0.39 μM, 0.78 μM, 1.56 μM, 3.13 μM, 6.25 μM, 12.5 μM, 25 μM, 50 μM, 100 μM and 200 μM were used to detect whether the inhibitory effect of compound Hit 1 on MRGPRX4 was concentration-dependent. As shown in Figure 9c, the percentage inhibition of DCA-induced Ca^2+^ signaling increased with increasing Hit 1 concentration, and its IC50 was 2.66 μM (95% CI: 1.324 to 4.958 μM; R^2^ = 0.624) when fitting with a variable slope model.

### 2.6. MRGPRX4 and 6′-O-Malonyldaidzin Binding Potential

DARTS (Drug Affinity Responsive Target Stability) can assess the interaction between a drug and its target protein. In this study, HEK293 cells stably expressing MRGPRX4 were treated with 50 μM 6′-O-malonyldaidzin or 0.01% DMSO for 3 h, followed by proteolysis with Pronase at mass ratios of 1:300, 1:600, and 1:900. Western blot analysis showed that 6′-O-malonyldaidzin treatment partially protected MRGPRX4 from protease degradation, as indicated by a relatively stronger FLAG-tagged band intensity compared with the DMSO control (Figure 9d). Densitometric quantification of band intensities from three independent experiments revealed that 6′-O-malonyldaidzin significantly increased MRGPRX4 stability, suggesting the presence of binding.

CETSA (Cell-based Thermal Shift Assay) is an effective strategy to detect the interaction between small molecules and protein targets in cells. Proteins are degraded with increasing temperature, but proteins bound to small molecules are relatively preserved at the same temperature. As shown in Figure 9e, MRGPRX4 protein expression decreased with increasing temperature from 37 °C to 62 °C. Pretreatment with 6′-O-malonyldaidzin slowed down this degradation trend, indicating that 6′-O-malonyldaidzin improved the thermostability of MRGPRX4 in intact cells. Densitometric analysis of three independent CETSA experiments showed a statistically significant thermal shift (*p* < 0.05). DARTS and CETSA results mutually support the direct engagement of 6′-O-malonyldaidzin with MRGPRX4, consistent with target binding.

## 3. Discussion

Cholestatic pruritus remains one of the most refractory symptoms in hepatobiliary disease, severely compromising patient quality of life. Current pharmacotherapies, including ursodeoxycholic acid, cholestyramine, and rifampicin, provide only partial relief in a subset of patients, highlighting the urgent need for mechanism-based therapeutic strategies. The identification of MRGPRX4 as a bile acid-responsive receptor predominantly expressed in human dorsal root ganglion neurons has opened a promising avenue for targeted intervention in cholestatic itch. However, the development of selective MRGPRX4 antagonists remains in its infancy, and no small-molecule inhibitor has yet advanced to clinical evaluation.

In the present study, we employed a structure-based virtual screening strategy to identify candidate MRGPRX4 antagonists from a natural product-enriched compound library comprising 13,144 entries in the TCMSP database. A hierarchical docking protocol (HTVS–SP–XP) was combined with MM/GBSA binding free energy rescoring, after which the final five hits were prioritized on the basis of four criteria: favorable Glide XP docking score, favorable MM/GBSA binding free energy, commercial availability, and structural diversity. The docking protocol was further validated by redocking the co-crystallized ligand from PDB 8K4S, which reproduced the experimental pose with a heavy-atom RMSD of 2.7 Å. Among them, 6′-O-malonyldaidzin emerged as the most promising candidate based on converging computational and experimental evidence.

From a computational standpoint, 6′-O-malonyldaidzin exhibited the most favorable binding free energy (ΔG = −75.15 kcal/mol) and formed a multi-point interaction network with key residues in the MRGPRX4 orthosteric pocket, including hydrogen bonds with ARG86, ARG95, and LEU96, as well as π–π stacking interactions with TRP158 and TYR240. These residues constitute a positively charged pocket flanked by aromatic side chains, an architecture that is consistent with the recognition of negatively charged bile acid substrates such as DCA and UDCA. The engagement of both electrostatic and hydrophobic interaction modalities by 6′-O-malonyldaidzin suggests a competitive antagonism mechanism at the bile acid-binding site, although definitive confirmation will require mutagenesis studies of the key interacting residues.

Molecular dynamics simulations over the 50 ns trajectory further corroborated the stability of the 6′-O-malonyldaidzin–MRGPRX4 complex. This compound did not exhibit the lowest protein Cα RMSD among the five complexes (folinic acid: 2.39 Å vs. 6′-O-malonyldaidzin: 2.73 Å); however, 6′-O-malonyldaidzin showed the smallest SASA value (14,634.08 Å^2^) and well-maintained Rg values within the 19–20 Å range, collectively indicating a compact and conformationally stable binding mode. Free energy landscape analysis revealed a well-defined low-energy basin, reinforcing the thermodynamic favorability of this complex. In contrast, while adenosine triphosphate and folinic acid also showed acceptable RMSD values, their high polarity and solvation penalties likely diminish cellular membrane permeability and effective target occupancy, which may explain their relatively modest functional activity despite reasonable computational metrics. This observation underscores an important principle in virtual screening: binding stability in silico does not necessarily translate into functional antagonism in cellulo, and orthogonal experimental validation remains indispensable. We note that these observations are limited to the 50 ns simulation timescale and do not establish long-term complex stability.

In silico ADMET profiling using ADMETlab 3.0 [25] indicated that 6′-O-malonyldaidzin possesses a potentially favorable pharmacokinetic profile for lead compound development based on computational predictions. Its predicted human intestinal absorption (HIA ≥ 30%), moderate plasma protein binding (75.05%), adequate hepatic microsomal stability (>30 min), and half-life of approximately 2.55 h suggest potentially reasonable oral bioavailability and sustained systemic exposure. Furthermore, 6′-O-malonyldaidzin exhibited the lowest overall predicted toxicity score among the five candidates, with only low-level predicted risks for AMES mutagenicity and hERG channel blockade. The absence of predicted blood–brain barrier penetration is a particularly desirable attribute for a peripherally acting antipruritic agent, as it would minimize the potential for central nervous system-related adverse effects. Nonetheless, these computational predictions require validation through in vitro metabolic stability assays (e.g., Caco-2 permeability, hepatocyte metabolism), plasma protein binding measurements, and formal in vivo pharmacokinetic studies before drawing definitive conclusions regarding drug-like properties.

Functionally, 6′-O-malonyldaidzin potently inhibited DCA-induced Ca^2+^ signaling in HEK293 cells stably overexpressing MRGPRX4, achieving 91% inhibition at 50 μM with a calculated IC50 of 2.66 μM. This level of potency is notable for a natural product-derived hit compound identified through virtual screening without prior structural optimization. The dose-dependent inhibition profile and the magnitude of the IC_50_ value suggest that 6′-O-malonyldaidzin merits further medicinal chemistry efforts to improve potency and selectivity. Importantly, 6′-O-malonyldaidzin showed minimal cross-reactivity with MRGPRX2, a related receptor implicated in mast cell-mediated itch and drug-induced pseudo-allergic reactions. Relatively selective profile is encouraging, as off-target modulation of MRGPRX2 could elicit undesirable immunological side effects. Nevertheless, a comprehensive selectivity panel encompassing additional MRGPR family members and other GPCRs will be necessary to fully characterize the pharmacological specificity of this compound.

The physical interaction between 6′-O-malonyldaidzin and MRGPRX4 was supported by two complementary biophysical approaches. DARTS analysis demonstrated enhanced resistance of MRGPRX4 to protease digestion upon compound treatment, while CETSA revealed a thermal stabilization shift in the receptor in intact cells. Densitometric quantification from three independent experiments confirmed a statistically significant protective effect (*p* < 0.05). The concordance between these orthogonal assays provides supportive evidence for direct target engagement at the cellular level. When considered together with the functional Ca^2+^ data, these findings suggest a coherent link between physical binding and downstream signaling inhibition, although definitive confirmation of the binding mode requires further structural studies.

Several limitations of the current study should be acknowledged. First, the virtual screening was confined to compounds within the TCMSP database, which primarily represents natural products from traditional Chinese medicine. While this library is rich in structural diversity, expanding the search to synthetic compound libraries may yield antagonists with superior potency or drug-like properties. Second, the functional assays were performed exclusively in an overexpression cell system (HEK293-MRGPRX4), which does not fully recapitulate the physiological context of human sensory neurons. Therefore, 6′-O-malonyldaidzin should be considered a promising lead compound for further pharmacological characterization and lead optimization, rather than an immediate therapeutic agent for cholestatic pruritus at the present stage. The use of human-induced pluripotent stem cell-derived sensory neurons or primary dorsal root ganglion cultures would provide a more translationally relevant assessment of compound activity. Third, the lack of a murine ortholog of MRGPRX4 with conserved bile acid responsiveness precludes conventional in vivo validation in rodent models of cholestatic pruritus [27]. Future studies should consider humanized mouse models or ex vivo human skin–nerve preparations to evaluate the antipruritic efficacy of 6′-O-malonyldaidzin in a more physiologically relevant setting. Fourth, while DARTS and CETSA confirmed target engagement, the precise binding mode and the molecular determinants of antagonism remain to be elucidated through site-directed mutagenesis and, ideally, co-crystallography or cryo-EM structural studies of the MRGPRX4–6′-O-malonyldaidzin complex.

In summary, this study presents an integrated computational and experimental workflow that identified 6′-O-malonyldaidzin as a promising lead compound targeting MRGPRX4 with low-micromolar potency, supported cellular target engagement, and a potentially favorable pharmacokinetic and safety profile based on in silico predictions. These findings provide a foundation for further pharmacological characterization, structure–activity relationship studies, and lead optimization efforts aimed at the eventual development of novel therapeutics for cholestatic pruritus.

## 4. Materials and Methods

### 4.1. Drugs and Reagents

Ganoderic acid C6 (Cat# CFN92290), 6′-O-malonyldaidzin (Cat# CFN91028) and 4-caffeoylquinic acid (Cat# CFN99117) acid were all purchased from Chem Faces (Wuhan, China). Folinic acid (Cat# F8983D48D) and adenosine triphosphate (Cat# A8833D48D) were all purchased from Jizhi Biochemical (Shanghai, China). Deoxycholic acid (Cat# T2965), ursodeoxycholic acid (Cat# T0700) and U73122 (Cat# T6243) were all purchased from TargetMol (Shanghai, China). Fluo-4, AM (Cat# PMK0933) was purchased from Bioprimacy (Wuhan, China). BASIC HBSS (Cat# C05-03020) was purchased from Gibco (Suizhou and China). 1 M HEPES Solution (Cat# BL2188A) was purchased from Biosharp (Jiangsui and China). Pluronic F-127 (Cat# D50421) was purchased from Bioss (Beijing, China). DYKDDDDK Tag (FLAG) Mouse Monoclonal Antibody (Cat# M1403-2) was purchased from HUABIO (Hangzhou and China).

### 4.2. Virtual Screening

MRGPRX4 (PDB ID: 8K4S, resolution: 2.90 Å) from the Protein Data Bank16 (PDB, http://www.rcsb.org/ (accessed on 8 September 2025)). A total of 13,144 compounds were obtained from the TCMSP database (https://www.tcmsp-e.com/tcmsp.php (accessed on 10 September 2025)). Ligand–protein docking was performed using Glide software (Schrodinger, LLC, New York, NY, USA).

Preprocessing before virtual screening: (1) Use the LigPrep module in Schrödinger Maestro 11.5 to prepare the ligands for the compounds, including generating a reasonable three-dimensional structure, predicting and optimizing the ionization state of the compounds under the condition of pH 7.0 ± 2.0, eliminating geometric conflicts, and assigning the OPLS_2005 force field to optimize the conformation and energy of the ligands17. (2) Use the Protein Preparation Wizard module to preprocess the protein, including hydrogenation, optimization of hydrogen bond networks, protonation under the condition of pH 7.0 ±2.0, and removal of water molecules, and finally use the OPLS_2005 force field to minimize the energy of the protein for optimizing the structure and eliminating geometric inappropriateness. (3) Use the Receptor Grid Generation module to define the active site residues of the original ligand in the MRGPRX4 structure and generate the receptor grid. Use the Ligand Docking module in Schrödinger Maestro 11.5 to perform Glide docking for the compounds. Successively use High-Throughput Virtual Screening (HTVS), Standard Precision (SP), and Extra Precision (XP) for molecular docking, and select docking score < −5 kcal/mol and the top 10% compounds for the next round of calculation. The top 5 commercially available compounds filtered by Glide XP are used for the next analysis, including molecular dynamics simulation and binding free energy calculation (MM/GBSA)

### 4.3. Prediction of ADMET and Physicochemical Properties of Candidate Compounds

The ADMET properties of 5 compounds were predicted using the ADMETlab3.0 online tool, and their physicochemical properties were evaluated. The two-dimensional structures of each compound were input into the ADMEtlab3.0 platform to obtain parameters related to drug development, including absorption, distribution, metabolism, excretion, and toxicity indicators, which were used for the preliminary evaluation of the pharmacological characteristics of the candidate compounds.

At the same time, the physicochemical property parameters—such as relative molecular mass, lipid–water partition coefficient, number of hydrogen bond donors, and number of hydrogen bond acceptors—of the 5 compounds were predicted and compared. Combined with the ADMET prediction results and physicochemical property parameters, the drug-likeness and further structural optimization potential of the 5 compounds were comprehensively evaluated.

### 4.4. Construction of the MRGPRX4 Overexpression Vector and Establishment of Stable Cell Lines

The human MRGPRX4 [28,29] coding sequence (NM_054032.3) was amplified by PCR and cloned into a linearized expression vector digested with BamHI/XhoI using seamless cloning. The recombinant plasmid, pSE8284, was transformed into DH5α competent cells, and positive clones were screened by colony PCR and confirmed by Sanger sequencing. The verified plasmid was then used to infect HEK293 cells, together with the control lentiviral vector pMT860. Forty-eight hours after infection, cells were selected with puromycin (2 μg/mL) for 2 weeks to generate a stable HEK293 cell line overexpressing MRGPRX4.

### 4.5. Western Blot and QPCR

HEK293 cells infected with the control lentivirus pMT860 or the MRGPRX4-overexpressing lentivirus pSE8284 were used to evaluate MRGPRX4 expression. For quantitative real-time PCR, MRGPRX4 mRNA levels were detected using β-actin as the internal control, and relative expression was calculated using the 2-ΔΔCt method. For Western blotting, total proteins were extracted from cells, quantified using a BCA assay, separated by SDS-PAGE, and transferred onto PVDF membranes. FLAG-tagged MRGPRX4 was detected using an anti-FLAG primary antibody and an HRP-conjugated anti-mouse IgG secondary antibody, followed by ECL visualization.

The primers used were as follows (Table 3):

### 4.6. Inhibitory Effects of Candidate Compounds on MRGPRX4

HEK293 cells stably expressing MRGPRX4 were seeded at a density of 2 × 10^5^/mL onto 96-well black-bottomed transparent-bottomed culture plates coated with poly-D-lysine solution, with 100 μL per well. The cells were cultured in a 37 °C, 5% CO_2_ incubator overnight. After approximately 24 h, the culture medium in the wells was discarded, and the cells were gently washed twice with Flex buffer (containing Ca^2+^, Mg^2+^, Na^+^, pH 7.4). The Flex buffer was removed, and then 50 μL of Fluo-4 buffer (containing 4 μM Fluo-4, AM, pH 7.4) was added. The cells were incubated in the dark at 37 °C for 60 min. Then, the dye working solution was discarded, and the cells were gently washed twice with Flex buffer, and 50 μL of the compound at twice the concentration dissolved in Flex buffer was added to each well. The cells were incubated at room temperature for 20 min. Next, the agonists DCA (deoxycholic acid, 10 μM) and UDCA buffer (ursodeoxycholic acid 10 μM) were placed in a regular 96-well plate, with 200–300 μL placed in each well. Calcium flux detection was performed using the Flex Station 3 instrument, with the excitation wavelength set at 490 nm and the emission wavelength at 525 nm. After 20 s from the baseline, 50 μL of the agonist DCA buffer was automatically added and the fluorescence value change was recorded. The measurement was continuously performed for 90 s. The test was conducted with 3 replicates, and the signal was averaged. The response was determined by subtracting the minimum signal (the baseline before adding the drug) from the maximum signal (obtained after drug stimulation). The inhibition rate is calculated according to Formula (1). The dose–response curve was plotted using the variable slope model curve fitting. The dose–response curve fitting and EC50 calculation were performed using GraphPad Prism software (version 10.5). Each concentration was tested in 3 independent biological replicates, each with 3 technical replicates (i.e., 3 wells per concentration per experiment).

(1) $$\mathrm{Inhibition}\;\left(\%\right)=1-\frac{{F_{\mathrm{positive control}}-F}_{\mathrm{test compound}}}{{F_{\mathrm{positive control}}-F}_{\mathrm{negative control}}}$$

F_positive control_: fluorescence value of the inhibitor; F_negative control_: fluorescence value of the agonist.

### 4.7. DARTS and CETSA

Stable HEK293 cells expressing MRGPRX4 were inoculated into the culture flask. When the cells reached an 80% density, 0.01% DMSO and 50 μM Hit 1-containing DMEM medium (without fetal bovine serum) were added, respectively. After 3 h of culture, the cells were lysed with RIPA lysate containing protease and phosphatase inhibitors on ice and the protein was collected. The total protein concentration was determined by BCA kit and leveled. The samples in each group were equally divided into three parts, and the Pronase solution was added according to mass ratios of Pronase to protein of 1:300, 1:600, and 1:900, respectively. The enzyme cleavage reaction was carried out by incubation at room temperature for 30 min. Loading buffer was added. It was heated at 100 °C for 10 min, and then SDS-PAGE was performed. After SDS-PAGE, we analyzed the expression of FLAG via the Western blot method [30,31].

Cell treatment and protein extraction were consistent with the DARTS assay. The flask was then placed at 37 °C for further incubation for 3 h. After incubation, the cells were washed three times with PBS and lysed with the lysis buffer containing protease and phosphatase inhibitors, and the cells were scraped and collected after 30 min. The cells were centrifuged at 14,000 rpm for 20 min at 4 °C, and the supernatant was collected. One part of the supernatant after centrifugation was used for protein quantification, and the other part was evenly divided into 6 portions. It was heated at different temperatures (37 °C, 42 °C, 47 °C, 52 °C, 57 °C, 62 °C) for 10 min using a PCR instrument, then cooled to room temperature for 3 min. It was centrifuged again at 14,000 rpm for 20 min at 4 °C, and the supernatant was collected. Loading buffer was added. It was heated it at 100 °C for 10 min, and then SDS-PAGE was performed. After SDS-PAGE, we analyzed the expression of FLAG protein via the Western blot method [32,33]. Band intensities were quantified using ImageJ (version 1.52a). Data from at least three independent experiments were analyzed via two-way ANOVA. *p* < 0.05 was considered statistically significant.

### 4.8. Statistical Analysis

Pharmacological data (IC_50_, EC_50_) were analyzed using nonlinear regression (variable slope model, four-parameter logistic curve, abbreviated 4PL) in GraphPad Prism. Goodness of fit was assessed by R^2^. Data are presented as mean ± SEM. Comparisons between two groups were performed using two-tailed *t*-tests; one-way ANOVA with Dunnett’s post hoc test was used for multiple comparisons. *p* < 0.05 was considered significant.

## 5. Conclusions

In this study, we identified 6′-O-malonyldaidzin as a promising small-molecule antagonist of MRGPRX4 through an integrated structure-based virtual screening and experimental validation workflow. The compound exhibited favorable binding affinity, stable receptor interaction in molecular dynamics simulations, and potent inhibition of DCA-induced Ca^2+^ signaling in HEK293 cells overexpressing MRGPRX4. In addition, DARTS and CETSA assays supported its direct engagement with MRGPRX4, and preliminary in silico ADMET analysis indicated a potentially favorable pharmacokinetic and safety profile pending experimental validation. Collectively, these findings support 6′-O-malonyldaidzin as a promising lead compound with preliminary selectivity for MRGPRX4 over MRGPRX2, warranting further pharmacological characterization and lead optimization. These results provide a basis for the eventual development of novel mechanism-based therapeutics for cholestatic pruritus. Further studies on physiologically relevant models and the structural validation of the binding mechanism are warranted.

## Figures and Tables

**Table 1:** Absorption, distribution, metabolism, excretion and toxicity (ADMET) evaluation of compounds.

Compound	Absorption	Distribution	Metabolism	Excretion	Toxicity
	HIA	PPB	HLM Stability/min	T_1/2_/h	Rat Oral Acute Toxicity/mg·kg^−1^
6′-O-malonyldaidzin	≥30%	75.05%	>30	2.55	>500
4-Caffeoylquinic acid	≥30%	48.77%	>30	2.58	>500
Folinic acid	≥30%	69.28%	>30	1.12	>500
Ganoderic acid C6	≥30%	76.79%	>30	1.66	>500
Adenosine triphosphate	≥30%	28.09%	>30	1.99	>500

**Table 2:** QPCR was used to detect the Ct value.

Group	β-Actin Ct	MRGPRX4 Ct	Relative MRGPRX4 Expression
HEK293-pMT860	17.06/17.14	33.82/34.24	1.01
HEK293-pSE8284	17.40/17.21	20.37/20.53	14,200.05

**Table 3:** List of primer sequences.

Gene	Forward Primer	Reverse Primer
MRGPRX4	TTGCCATTACGCCTCATC	AAAGTAGGGAAAGGTCATCAC
β-actin	CATGTACGTTGCTATCCAGGC	CTCCTTAATGTCACGCACGAT

## Data Availability

The data presented in this study are available on request from the corresponding author.

## Abbreviations

The following abbreviations are used in this manuscript:

ADMET	Absorption, Distribution, Metabolism, Excretion and Toxicity
CETSA	Cell-based Thermal Shift Assay
DMEM	Dulbecco’s Modified Eagle Medium
DCA	Deoxycholic acid
DARTS	Drug Affinity Responsive Target Stability
FBS	Fetal Bovine Serum
HTVS	High-Throughput Virtual Screening
HIA	Human Intestinal Absorption
MM/GBSA	Binding Free Energy Calculation
SP	Standard Precision
UDCA	Ursodeoxycholic acid
XP	Extra Precision
